# Availability of Mental Telehealth Services in the US

**DOI:** 10.1001/jamahealthforum.2023.5142

**Published:** 2024-02-02

**Authors:** Jonathan Cantor, Megan S. Schuler, Samantha Matthews, Aaron Kofner, Joshua Breslau, Ryan K. McBain

**Affiliations:** 1 RAND Corporation, Santa Monica, California; 2 RAND Corporation, Boston, Massachusetts; 3 RAND Corporation, Arlington, Virginia; 4 RAND Corporation, Pittsburgh, Pennsylvania

## Abstract

**Importance:**

Telehealth utilization for mental health care remains much higher than it was before the COVID-19 pandemic; however, availability may vary across facilities, geographic areas, and by patients’ demographic characteristics and mental health conditions.

**Objective:**

To quantify availability, wait times, and service features of telehealth for major depressive disorder, general anxiety disorder, and schizophrenia throughout the US, as well as facility-, client-, and county-level characteristics associated with telehealth availability.

**Design, Settings, and Participants:**

Cross-sectional analysis of a secret shopper survey of mental health treatment facilities (MHTFs) throughout all US states except Hawaii from December 2022 and March 2023. A nationally representative sample of 1938 facilities were contacted; 1404 (72%) responded and were included. Data analysis was performed from March to July 2023.

**Exposure:**

Health facility, client, and county characteristics.

**Main Outcome and Measures:**

Clinic-reported availability of telehealth services, availability of telehealth services (behavioral treatment, medication management, and diagnostic services), and number of days until first telehealth appointment. Multivariable logistic and linear regression analyses were conducted to assess whether facility-, client-, and county-level characteristics were associated with each outcome.

**Results:**

Of the 1221 facilities (87%) accepting new patients, 980 (80%) reported offering telehealth. Of these, 97% (937 facilities) reported availability of counseling services; 77% (726 facilities), medication management; and 69% (626 facilities) diagnostic services. Telehealth availability did not differ by clinical condition. Private for-profit (adjusted odds ratio [aOR], 1.75; 95% CI, 1.05-2.92) and private not-for-profit (aOR, 2.20; 95% CI, 1.42-3.39) facilities were more likely to offer telehealth than public facilities. Facilities located in metropolitan counties (compared with nonmetropolitan counties) were more likely to offer medication management services (aOR, 1.83; 95% CI, 1.11-3.00) but were less likely to offer diagnostic services (aOR, 0.67; 95% CI, 0.47-0.95). Median (range) wait time for first telehealth appointment was 14 (4-75) days. No differences were observed in availability of an appointment based on the perceived race, ethnicity, or sex of the prospective patient.

**Conclusions and Relevance:**

The findings of this cross-sectional study indicate that there were no differences in the availability of mental telehealth services based on the prospective patient’s clinical condition, perceived race or ethnicity, or sex; however, differences were found at the facility-, county-, and state-level. These findings suggest widespread disparities in who has access to which telehealth services throughout the US.

## Key Points

**Question:**

What is the availability of different levels of telehealth services offered through mental health treatment facilities (MHTFs) across the US, and does availability differ by the client-caller’s demographic characteristics, mental health condition, or facility location?

**Findings:**

This cross-sectional secret shopper study of 1404 MHTFs conducted from December 2022 to March 2023 found that privately owned facilities with only outpatient services were most likely to offer telehealth services. No differences were found to be associated with the client-caller’s perceived race, ethnicity, sex, or presenting mental health condition.

**Meaning:**

These findings suggest that there are significant differences in the availability of telehealth appointments by location of MHTFs across the US, but few differences based on the client-caller’s demographic characteristics or mental health condition.

## Teaser

This cross-sectional secret shopper study evaluates the availability of mental telehealth availability across the US and its associations with patient demographic characteristics and facility location.

## Introduction

Telehealth utilization in the US expanded considerably during the COVID-19 pandemic,^1,2,3,4,5^ enabled by changes in federal and state policies pertaining to financial reimbursement of these services.^6,7^ This shift was particularly pronounced for mental health services: the number of outpatient treatment facilities providing telehealth^8^ and the proportion of visits that were conducted using telehealth rose substantially.^9,10,11^ Studies of commercial and public insurance claims have demonstrated that the overall volume of services for mental health conditions remained stable throughout the pandemic despite restrictions on in-person care. This stability has been largely attributed to the rise in telehealth visits.^9,12,13^

During the pandemic, telehealth utilization rose and then returned to nearly prepandemic levels in most fields of medicine; however, it remained much higher than prepandemic levels in mental health care.^11^ Although studies have evaluated utilization of telehealth throughout the pandemic, availability and composition of telehealth services remain largely undocumented including ease of access to appointments, mental health conditions treated, types of telehealth services offered, and accepted types of insurance coverage. Availability is the extent to which a facility has requisite resources to meet the needs of a client.^14^ Understanding availability of telehealth is important for informing policies that maximize the potential benefits of telehealth for mental health services.

This cross-sectional study evaluates the availability of telehealth services for mental health care using a secret shopper approach. In secret shopper studies, researchers contact facilities posing as prospective clients who are directly inquiring about service availability, thus mitigating potential social desirability bias that could be generated from a formal research survey.^15^ Although secret shopper approaches have been used previously to assess the availability of mental health treatment,^16,17,18^ to our knowledge none have focused on telehealth services. Based on the rapid scale-up of telehealth over the past 3 years, a secret shopper analysis provides a clear and timely picture of service composition throughout the US. Using a nationally representative sample of outpatient mental health facilities (MHTFs), we evaluated service composition and facility, client characteristics, and geographic differences in availability of telehealth. Given the variations among US states’ telehealth coverage policies and reimbursements environments, we expected to observe heterogeneity in availability of telehealth services across states.

## Methods

Our research team contacted 1938 MHTFs between December 2022 and March 2023 using a standardized client script to inquire about current facility telehealth availability. The focus was on MHTFs that provided treatment to adults. We collated secret shopper survey responses alongside facility- and county-level characteristics. The combined data were used to understand correlates of telehealth availability at MHTFs across the US. The study was approved by the RAND Corporation’s Human Subjects Protection Committee, and informed consent was waived because the study did not constitute human subjects research. This study followed the Strengthening the Reporting of Observational Studies in Epidemiology (STROBE) guidelines.

### Sampling

Our sampling frame was defined as outpatient MHTFs throughout the US, as reported in the RAND Corporation’s Mental Health and Addiction Treatment Tracking Repository (MATTR) according to the Behavioral Health Treatment Service Locator (Substance Abuse and Mental Health Services Administration). The locator data are informed by the annual US National Substance Use and Mental Health Services Survey. The survey collects data from all mental health treatment facilities in the US and includes specialty facilities identified by state mental health authorities. The sample includes data from psychiatric hospitals, general hospitals with a separate inpatient psychiatric unit, state hospitals, Veterans Affairs medical centers, certified community behavioral health clinics, daily partial hospitalization treatment facilities, outpatient facilities, and residential treatment centers. The data do not include individual private practitioners. The locator is updated monthly with new facilities, and individual facilities can update their data weekly. We abstracted facility address and telephone number for contact purposes. MATTR also contains data on whether the facility provides inpatient and/or outpatient services, accepted insurance types, facility type (community mental health center or other type), and whether it is public or private. We restricted the study sample to facilities that provide outpatient services because inpatient services are less conducive to the telehealth format.

Facility addresses were used to link to county-level data within the Health Resources & Services Administration’s Area Health Resource Files. Specifically, we collated information on county urbanicity, percentage of Black and/or Hispanic residents, percentage of residents older than 25 years with a high school education, and median household income.^19^

### Data Collection

We extracted data regarding 9568 MHTFs from the MATTR dataset as of August 22, 2022. These data included information from the previous year’s National Substance Use and Mental Health Services Survey. We excluded VA medical centers (n = 356), facilities with multiple separate programs at the same geographic location (n = 520; to avoid confusion regarding contacting the correct program), and facilities that did not offer any outpatient services (n = 1766); exclusions were not mutually exclusive.

From the remaining 7092 facilities, we randomly selected 1938 (27.3%) to attempt to contact in December 2022 through March 2023. We received responses from 1404 facilities (72.5%). The remaining 534 MHTF facilities (27.5%) could not be contacted and were excluded—255 (13.2%) did not answer the telephone after 3 calls; 128 (6.6%) were pediatric only; 62 (3.2%) were either permanently closed or the telephone number had been disconnected; and 88 (4.5%) were excluded for other reasons (eg, the facility was inpatient only or did not provide behavioral health services). A larger share of the responding facilities provided outpatient services only, were community mental health centers, and were more likely to accept private insurance compared with those that we failed to successfully reach. Facilities located in a metropolitan county or in counties with below median household incomes or below median high school graduation rates were more likely to respond. The race and ethnic distributions of counties in which responding and nonresponding facilities were located were also significantly different.

Trained callers used a standardized script—developed based on best practices from the secret shopper literature^16,20,21^—and posed as prospective clients with a mental health condition. Callers were randomly assigned to stating that they were seeking services for 1 of 3 clinical conditions: major depressive disorder (MDD), generalized anxiety disorder (GAD), and schizophrenia. To determine whether perceived client demographic characteristics were associated with telehealth availability, we randomized the name by which the callers identified themselves, using historically female and male names and Black, White, or Hispanic names. The specific names we used were selected from either a previous audit study^22^ or a combination of a separate audit study and name-frequency website based on registry data.^23,24^ By varying the callers’ perceived race, ethnicity, and sex, we sought to assess a form of linguistic profiling over the telephone.^25^ Callers then inquired about specific aspects of telehealth availability. Facility responses were recorded during each call using Qualtrics (Silver Lake). The full script and protocol can be found in the eAppendix in Supplement 1.

### Study Outcomes

The study’s primary outcome was the current availability of telehealth services at each facility (yes/no) based on staff responses to callers. For MHTFs that reported telehealth service availability, other measures of interest were whether services were offered via telehealth for specific clinical conditions (ie, MDD, GAD, schizophrenia), types of services offered (ie, behavioral therapy, medication management, diagnostic services), and number of days until the next available appointment.

### Statistical Analysis

We report facility- and county-characteristics of MHTFs that responded to the survey. Using χ^2^ testing, we compared them with MHTFs that were contacted but that did not respond. Among survey respondents, we conducted descriptive analyses to characterize features of telehealth services at MHTFs throughout the US. Additionally, we conducted multilevel logistic regression analysis to identify factors at the facility, client, and geographic level that were associated with telehealth availability. Similarly, we conducted multilevel linear regression analyses to identify factors associated with telehealth wait time; these models included state fixed effects. In all models, facility-level characteristics included whether the facility was outpatient only (compared with facilities that also offered hospital inpatient and/or residential services), accepted Medicaid, accepted private insurance, and whether the facility was a community mental health center. We also included the county-level sociodemographic characteristics that are historically associated with availability of mental health care to control for potential confounding,^26,27,28^ structured as categorical measures: county urbanicity (dichotomized as metropolitan vs not metropolitan), percentage of residents who identified as Black (4 groups: <5%, 5% to <10%, 10% to <20%, and ≥20%), the share of residents who identified as Hispanic (4 groups: <5%, 5% to <10%, 10% to <20%, and ≥20%), median household income (dichotomized by median split as <$58 235 or ≥$58 235), and percentage over 25 years with a high school degree (dichotomized by median split as <89.2% or ≥89.2%). We note that cut points for share of residents who were Black and Hispanic were selected to create interpretable categories that capture meaningful differences in the relative populations of Black and Hispanic residents across counties. Lastly, we included indicators for the client’s stated mental health condition (ie, MDD, GAD, or schizophrenia) and race and ethnicity.

All regression models clustered standard errors at the state level and included either state fixed effects (for wait time models) or random effects (for all other models). Data with missing observations for the covariates or outcome measures were excluded from analysis. Statistical tests were using 2-tailed, and *P* values < .05 were considered statistically significant. Data analyses were performed from March to July 2023 using Stata, version 189 (StataCorp LLC).

## Results

### Overall Telehealth Availability

In all, 1404 facilities were successfully contacted. Table 1 provides descriptive information on the MHTFs that responded to the survey and were included compared with MHTFs that did not respond to the survey (eTable 1 in Supplement 1).

**Table 1.** Comparison of Facility and County Characteristics Between Respondents and Nonrespondents

Characteristic	No. (%)		*P* value^a^
	Respondents (n = 1404)	Nonrespondents (n = 534)	
Facility characteristics			
Services offered			
Outpatient services only^b^	1277 (91.0)	429 (80.3)	<.001
Ownership			
Government	198 (14.1)	59 (11.1)	.17
Private, for-profit	319 (22.7)	118 (22.1)	
Private, not-for-profit	887 (63.2)	357 (66.9)	
Community mental health center	376 (26.8)	74 (13.9)	<.001
Accepts Medicaid	1290 (91.9)	479 (89.7)	.13
Accepts private insurance	1236 (88.0)	410 (76.8)	<.001
County characteristics			
Rurality			
Not metropolitan	385 (27.4)	89 (16.7)	<.001
Metropolitan	1019 (72.6)	436 (81.7)	
Share of Hispanic residents, %			
<5	473 (33.7)	127 (23.8)	<.001
5-10	336 (23.9)	139 (26.0)	
10-20	294 (20.9)	109 (20.4)	
>20	301 (21.4)	150 (28.1)	
Share of non-Hispanic Black residents, %			
<5	626 (44.6)	193 (36.1)	<.001
5-10	274 (19.5)	128 (24.0)	
10-20	230 (16.4)	109 (20.4)	
>20	274 (19.5)	95 (17.8)	
Median household income			
Below median ($58 235)	439 (31.3)	101 (18.9)	<.001
Above median	965 (68.7)	424 (79.4)	
Share of residents aged ≥25 y with high school degree			
Below median (89.2%)	624 (44.4)	197 (36.9)	<.001
Above median	780 (55.6)	328 (61.4)	
Caller characteristics			
Mental health condition			
General anxiety disorder	477 (34.0)	NA	NA
Major depressive disorder	450 (32.1)	NA	
Schizophrenia	477 (34.0)	NA	
Perceived race and ethnicity		NA	
Hispanic	459 (32.7)	NA	
Non-Hispanic Black	488 (34.8)	NA	
Non-Hispanic White	457 (32.6)	NA	

Abbreviation: NA, not applicable.

^a^ Statistical testing was performed using χ^2^ tests.

^b^ Reference group is composed of facilities that offer hospital inpatient and/or residential services.

Facility characteristics come from a data download of the Mental Health and Addiction Treatment Tracking Repository dataset on August 22, 2022. County-level characteristics come from the 2020 American Community Survey. County-level characteristics percentages do not sum to 100 for nonrespondents because we lacked county-level characteristic for 9 facilities located in Puerto Rico or Northern Mariana Islands. Facilities were excluded if they did not offer any outpatient services, were operated by the US Department of Veteran Affairs, or had multiple colocated treatment programs.

Of 1221 facilities (87%) that were accepting new clients, 980 (80%) reported offering telehealth services (henceforth referred to as current telehealth facility). Overall, 47% of current telehealth facilities reported that their telehealth appointments were available via video (n = 573); 5% via audio (n = 49); and 47% via both video and audio (n = 573); 1% did not know the modality offered. In multivariable regression models, we did not find evidence that telehealth availability among MHTF facilities differed significantly according to the clinical condition for which telehealth services were sought. Reported availability for GAD was 79.6% (315 facilities); for MDD, 81.4% (316 facilities); and for schizophrenia, 83.1% (349 facilities).

**Figure 1.**
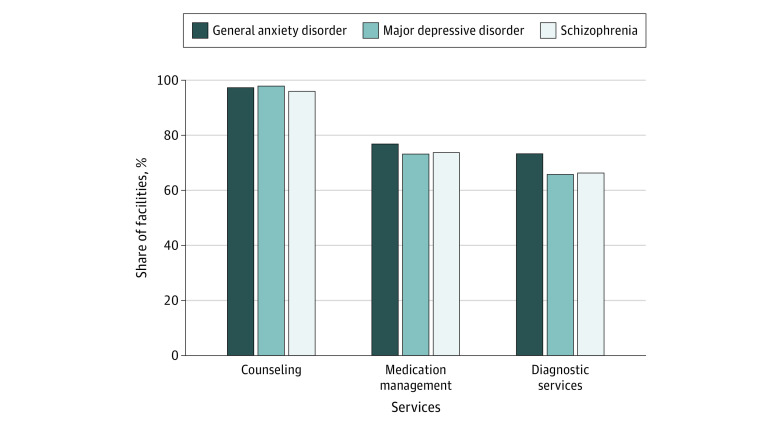
Types of Telehealth Services Offered at Mental Health Treatment Facilities Surveyed by Secret Shopper Client Condition Reported predicted probabilities obtained from logistic regression models that adjusted for client, facility, and county characteristics as well as state-level random effects.

There was wide variation at the state level in the proportion of MHTFs that were currently offering telehealth services (Figure 1**)**. For example, we found that less than half of MHTFs in Mississippi and South Carolina were offering telehealth services. In contrast, all MHTFs contacted in Delaware, Maine, New Mexico, and Oregon were offering telehealth services.

Among current telehealth facilities, 96.9% (937 facilities) reported availability of counseling services via telehealth; 76.7% (726 facilities), medication management; and 68.7% (626 facilities) diagnostic services. Responses did not differ significantly according to the stated clinical condition of the caller.

In logistic regression analysis, we found that facilities that provided only outpatient services (compared with facilities also offering hospital inpatient and/or residential services) were more likely to offer telehealth (adjusted odds ratio [aOR], 3.76; 95% CI, 2.37-5.99). In addition, private for-profit (aOR, 1.75; 95% CI, 1.05-2.92) and private not-for-profit (aOR, 2.20; 95% CI, 1.42-3.39) facilities were more likely to offer telehealth compared with public facilities. No county-level characteristics were predictive of whether a facility offered telehealth (Table 2).

**Table 2.** Logistic Regression Predicting Mental Telehealth Services Availability and Mental Telehealth Service Type

Characteristic	Mental telehealth services^a^			
	Available^b^	Service type		
		Counseling	Medication management	Diagnostics
Facility-respondent, No.	1342	1076	1059	1020
Caller-client characteristics				
Mental health condition; generalized anxiety disorder [Reference]				
Major depressive disorder	1.14 (0.85-1.52)	1.31 (0.41-4.21)	0.80 (0.53-1.22)	0.70 (0.49-0.99)^c^
Schizophrenia	1.35 (0.94-1.94)	0.66 (0.26-1.68)	0.83 (0.58-1.18)	0.71 (0.50-1.01)
Perceived race and ethnicity; non-Hispanic White [Reference]				
Black	0.81 (0.60-1.10)	1.71 (0.75-3.89)	1.01 (0.64-1.60)	0.87 (0.64-1.18)
Hispanic	0.98 (0.65-1.48)	1.16 (0.47-2.87)	1.03 (0.71-1.49)	0.97 (0.73-1.28)
Facility characteristics				
Accepts Medicaid payment	1.81 (0.98-3.33)	4.27 (1.56-11.69)^d^	1.80 (0.97-3.36)	0.69 (0.44-1.10)
Accepts private insurance payment	1.24 (0.72-2.12)	0.88 (0.35-2.20)	1.64 (1.00-2.68)^c^	0.83 (0.59-1.18)
Community mental health center	0.95 (0.64-1.41)	0.94 (0.33-2.66)	1.03 (0.63-1.67)	0.92 (0.64-1.32)
Ownership, US government [Reference]				
Private, for-profit	1.75 (1.05-2.92)^c^	1.09 (0.28-4.28)	0.31 (0.16-0.60)^b^	2.06 (1.28-3.31)^d^
Private, not-for-profit	2.20 (1.42-3.95)^b^	1.34 (0.43-4.12)	0.64 (0.34-1.20)	1.48 (0.97-2.27)
Outpatient facility; inpatient and outpatient [Reference]	3.77 (2.37-5.99)^b^	2.15 (0.61-7.65)	0.67 (0.38-1.19)	1.01 (0.60-1.68)
County characteristics				
Metropolitan; not metropolitan [Reference]	0.75 (0.52-1.09)	0.85 (0.31-2.36)	1.83 (1.11-3.00)^c^	0.67 (0.47-0.95)^c^
Share of Hispanic residents; <5% [Reference]				
5%-10%	1.00 (0.69-1.46)	1.17 (0.40-3.38)	0.95 (0.66-1.37)	1.39 (0.95-2.04)
10%-20%	1.01 (0.62-1.65)	1.10 (0.38-3.15)	0.97 (0.58-1.61)	1.76 (1.18-2.62)^d^
>20%	1.15 (0.72-1.83)	0.82 (0.17-3.87)	1.18 (0.71-1.97)	2.06 (1.40-3.03)^b^
Share of non-Hispanic Black residents; <5% [Reference]				
5%-10%	0.86 (0.50-1.46)	0.67 (0.29-1.58)	0.90 (0.57-1.40)	0.93 (0.56-1.54)
10%-20%	0.89 (0.53-1.50)	0.68 (0.28-1.62)	1.06 (0.63-1.78)	0.83 (0.53-1.31)
>20%	0.81 (0.47-1.41)	1.32 (0.44-3.97)	0.82 (0.48-1.39)	0.89 (0.58-1.36)
Above median household income; below median [Reference]	1.01 (0.73-1.40)	1.24 (0.34-4.46)	0.87 (0.59-1.27)	1.02 (0.66-1.58)
Above median % of residents with a high school degree; below median [Reference]	1.28 (0.90-1.81)	0.86 (0.27-2.76)	0.65c (0.42-1.00)	0.98 (0.66-1.47)

^a^ Each column is a separate regression model limited to the facility-respondents accepting new patients. Logistic regression was estimated to include state-level random effects. All coefficients and 95% CIs in parentheses are odds ratios. Standard errors were clustered at the state level. Differences in sample size reflect skip patterns in survey.

^b^ *P* < .001.

^c^ *P* < .05.

^d^ *P* < .01.

### Telehealth Services Availability

Logistic regression analysis indicated that facilities that reported accepting Medicaid as a form of payment were significantly more likely to offer counseling services via telehealth (aOR, 4.27; 95% CI, 1.56-11.69) compared with facilities that did not accept Medicaid. Facilities located in metropolitan areas (aOR, 1.83; 95% CI, 1.11-3.00) and facilities that accepted private insurance (aOR, 1.64; 95% CI, 1.00-2.68) were significantly more likely to offer medication management via telehealth. Private for-profit facilities (aOR, 0.30; 95% CI, 0.38-0.60) were significantly less likely to offer medication management via telehealth compared with public facilities. In contrast, private for-profit facilities (aOR, 2.06; 95% CI, 1.28-3.31) were more likely to offer diagnostic services via telehealth compared with public facilities.

**Figure 2.**
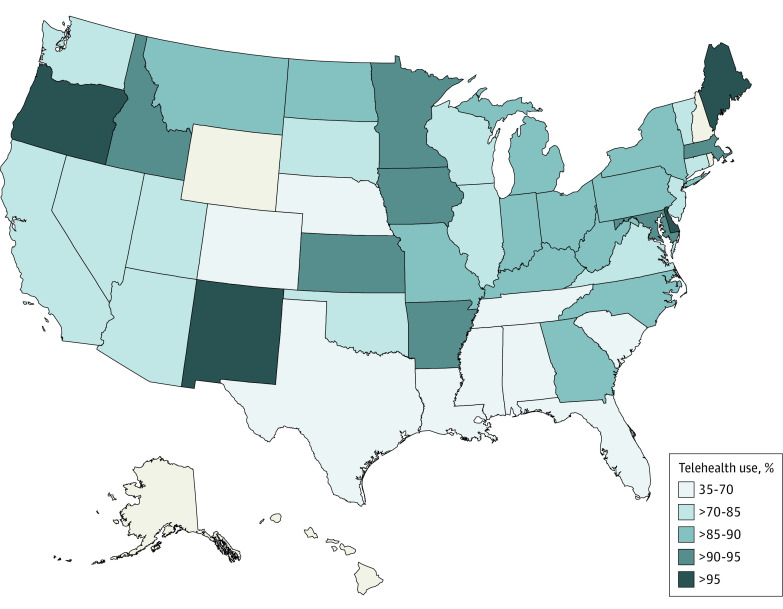
State-Specific Proportions of Surveyed Mental Health Treatment Facilities With Reported Telehealth Services (n = 1342) Share of facilities within a state that offered telehealth services. States in gray have less than 5 surveys administered; none were surveyed in Hawaii.

Regarding county-level characteristics, we found that facilities located in metropolitan counties were significantly less likely to offer diagnostic services via telehealth (aOR, 0.67; 95% CI, 0.47-0.95). Additionally, we found that clinics were significantly less likely to report availability of diagnostic services via telehealth when callers inquired about services for MDD compared with callers with GAD (aOR, 0.70; 95% CI, 0.49-0.99) (Table 2). Regression adjusted percentages for each of the telehealth services and mental health conditions are reported in Figure 2.

### Telehealth Wait Times

**Figure 3.**
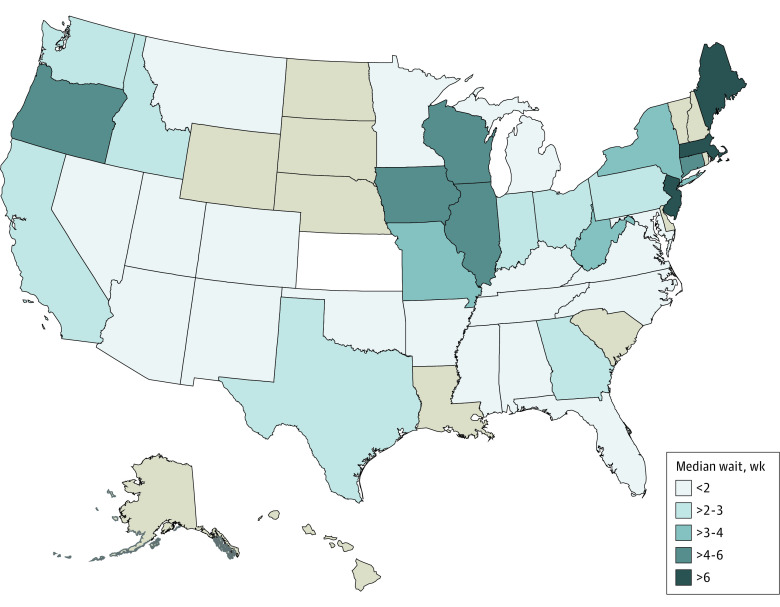
State-Specific Median Wait Times to Next Available Telehealth Appointment at Surveyed Mental Health Treatment Facilities (n = 598) Median number of weeks to the next available appointment from the date of the call to the facility. States in beige have fewer than 5 surveys administered; none were surveyed in Hawaii.

We also inquired about wait time for an initial telehealth appointment among current telehealth facilities (eTable 2 in Supplement 1). The median (IQR) number of days until first appointment was 14.0 (5-36) days. The number of days until the first available appointment did not differ according to clinical condition, caller, facility, or county characteristics (regression results not shown). However, we found broad differences in median telehealth wait times across states, as shown in Figure 3. The state with the longest median wait time for a telehealth appointment was Maine (75 days), and the state with the shortest median wait time for telehealth was North Carolina (4 days).

## Discussion

The COVID-19 pandemic has been associated with increased availability and utilization of mental telehealth services.^3,9,12^ This secret shopper survey provides insights regarding the experience of a typical client seeking specialty care from a MHTF in the US. Concerningly, approximately 1 in 5 facilities that we attempted to contact did not respond despite multiple attempts. This suggests that many individuals seeking a specialty mental health service may encounter difficulty in inquiring about treatment services. Among MHTF facilities that were successfully contacted, most (87% and 80%, respectively) were accepting new clients and were currently offering telehealth services. Telehealth availability varied across states, with fewer than half of all contacted MHTFs in Mississippi and South Carolina offering telehealth services compared with all MHTFs in several other states.

These findings also highlighted heterogeneity in both the types of clinical services that MHTFs offered via telehealth, as well as the modality through which telehealth was offered. Although almost all facilities offering telehealth included virtual counseling services, approximately 1 in 4 did not offer virtual medication management, and approximately 1 in 3 did not offer virtual diagnostic services. Most (94%) telehealth facilities offered telehealth via video appointments, whereas fewer (52%) offered audio-only appointments. Some possibilities for this may be that video appointments are considered to be more clinically effective^29,30,31^ and/or are preferred by patients.^32,33^ More work is needed to understand possible factors associated with video and telephone appointment availability.

Additionally, these study findings indicated that the median wait time for a telehealth appointment was more than 2 weeks (15 days), with significant geographic variation ranging from more than 2 months at MHTFs in Maine to 4 days at MHTFs in North Carolina. The typical wait time, nationally, for an in-person mental health appointment has not been well characterized. However, a recent survey of psychologists found that approximately 60% had no openings for new patients in 2021.^34^ Taken together, these findings underscore potential heterogeneity in geographic availability of treatment from specialty practitioners at MHTFs in the US. Possible reasons for the differences in telehealth appointment wait times could be regional differences in broadband internet access, whether MHTFs are experiencing a shortage of staff, and existing state-level telehealth policies. More work is needed to investigate the possible reasons for these differences. A potential vehicle to improve telehealth wait times would be to broaden policies allowing facilities to offer telehealth across state lines.

We did not observe differences in the availability of services based on perceived racial and ethnic identity or stated clinical condition of the caller. Although our findings do not suggest differences by perceived racial and ethnic identity at the individual level, we note that different racial and ethnic groups may experience differential treatment access due to residential segregation patterns in the US. Previous research found that telehealth services were less likely to be offered by MHTFs located in counties with a larger share of Black residents.^3^ However, we did observe differences in availability of telehealth according to facility ownership: private facilities were nearly twice as likely to offer telehealth services compared with public facilities. Interestingly, private for-profit facilities were much less likely to offer medication management via telehealth compared with public facilities; however, they were more than twice as likely to offer diagnostic services. One explanation for this is that public and private facilities tend to serve different populations. Prior to the COVID-19 pandemic, studies observed that public facilities were more likely to offer telehealth services,^35^ and we see a marked departure from this during the course of the pandemic, which may be associated with payment parity requirements of commercial health insurers.^3^

Policymakers have shared concern that rapidly evolving health care delivery modalities, including telehealth, could increase existing disparities in mental health care.^36^ The findings of this secret shopper survey are encouraging insofar as we did not observe a systematic bias in which available services differed based on perceived race and ethnicity of the client, stated clinical condition of the client, or county-level sociodemographic characteristics. However, our findings indicate that a prospective client may face several hurdles finding a facility that offers comprehensive telehealth services. Specifically, the patient must successfully contact the facility, confirm that the facility is accepting new patients and that it accepts the patient’s insurance, and must identify a facility that offers the specific telehealth services for their mental health needs. Our sampling frame uses administrative data to identify facilities. Given that 21% (n = 405) of the facilities did not answer the telephone call when we attempted to contact them is concerning for those seeking a specialty mental health practitioner.

### Limitations

This study had several limitations. First, our secret shopper survey focused on MHTFs; it is possible that telehealth availability and services may differ at other types of facilities. We note that the proportion of mental health treatment services provided through MHTFs compared with other types of facilities is not currently well characterized. Second, we did not directly measure telehealth utilization or quality, only availability. Third, our study was cross sectional; changes in telehealth-related policies, especially after the Public Health Emergency, may have shifted telehealth availability. Fourth, there was imperfect concordance between the caller’s race and ethnicity and the name of the caller (the study’s primary measure of perceived race and ethnicity), which may have affected the findings. Fifth, all of our secret shopper callers initiated and conducted calls in English; as such, we were unable to assess potential differential availability based on caller language. We note that linguistic profiling (reflecting potential bias and discrimination) is a key area for future research regarding mental health treatment access.^25^ Sixth, our findings that caller sociodemographic characteristics were not associated with clinic responses should be interpreted in the context of a small sample size and large confidence intervals regarding these estimates. Seventh, the MATTR dataset did not contain information on the number or types of clinicians at facilities. Lastly, we did not include measures of broadband coverage as a covariate. Future work should evaluate whether availability of telehealth appointments varies according to the quality of broadband internet service available at the facility location.

## Conclusions

This nationally representative secret shopper study of MHTFs found considerable variation in the types of telehealth services offered: one-third of facilities did not provide diagnostic services via telehealth and one-quarter did not provide medication management via telehealth. We also found significant differences across states in telehealth availability and average wait times for care. However, we did not observe significant differences in the availability of telehealth services based on county-level characteristics or the caller’s perceived race and ethnicity or mental health condition.

## Notes

Supplement 1. **eAppendix.** Secret Shopper Case Profiles and Survey Questionnaires **eTable 1.** Comparison of Facility and County Characteristics Between Successfully Contacted and Not Contacted Facilities **eTable 2.** Ordinary Least Squares Regression Predicting Wait Times

Supplement 2.
Data Sharing Statement

## References

[1] Mehrotra A, Nimgaonkar A, Richman B. Telemedicine and medical licensure: potential paths for reform. N Engl J Med. 2021;384(8):687-690. doi:10.1056/NEJMp2031608 33626604

[2] Mehrotra A, Bhatia RS, Snoswell CL. Paying for telemedicine after the pandemic. JAMA. 2021;325(5):431-432. doi:10.1001/jama.2020.25706 33528545 PMC9320940

[3] McBain RK, Schuler MS, Qureshi N, . Expansion of telehealth availability for mental health care after state-level policy changes from 2019 to 2022. JAMA Netw Open. 2023;6(6):e2318045. doi:10.1001/jamanetworkopen.2023.18045 37310741 PMC10265313

[4] Cantor JH, McBain RK, Ho PC, Bravata DM, Whaley C. Telehealth and in-person mental health service utilization and spending, 2019 to 2022. JAMA Health Forum. 2023;4(8):e232645. doi:10.1001/jamahealthforum.2023.2645 37624614 PMC10457709

[5] Kalmin MM, Cantor JH, Bravata DM, Ho PC, Whaley C, McBain RK. Utilization and spending on mental health services among children and youths with commercial insurance. JAMA Netw Open. 2023;6(10):e2336979. doi:10.1001/jamanetworkopen.2023.36979 37787996 PMC10548294

[6] Center for Connected Policy. Federal Telehealth Laws. Published 2023. Accessed June 17, 2023. https://www.cchpca.org/federal/

[7] Chu RC, Peters C, Lew ND, Sommers BD. State Medicaid Telehealth Policies Before and During the COVID-19 Public Health Emergency. Office of the Assistant Secretary for Planning and Evaluation, US Department of Health and Human Services; 2021. https://aspe.hhs.gov/sites/default/files/documents/eb9e147935a2663441a9488e36eea6cb/medicaid-telehealth-brief.pdf

[8] Cantor J, McBain RK, Kofner A, Hanson R, Stein BD, Yu H. Telehealth adoption by mental health and substance use disorder treatment facilities in the COVID-19 pandemic. Psychiatr Serv. 2022;73(4):411-417. doi:10.1176/appi.ps.202100191 34407631 PMC10695271

[9] McBain RK, Cantor J, Pera MF, Breslau J, Bravata DM, Whaley CM. Mental health service utilization rates among commercially insured adults in the US during the first year of the COVID-19 pandemic. JAMA Health Forum. 2023;4(1):e224936. doi:10.1001/jamahealthforum.2022.4936 36607697 PMC9857246

[10] Rosen CS, Morland LA, Glassman LH, . Virtual mental health care in the Veterans Health Administration’s immediate response to coronavirus disease-19. Am Psychol. 2021;76(1):26-38. doi:10.1037/amp0000751 33119331

[11] Patel SY, Mehrotra A, Huskamp HA, Uscher-Pines L, Ganguli I, Barnett ML. Variation in telemedicine use and outpatient care during the COVID-19 pandemic in the United States. Health Aff (Millwood). 2021;40(2):349-358. doi:10.1377/hlthaff.2020.01786 33523745 PMC7967498

[12] Busch AB, Huskamp HA, Raja P, Rose S, Mehrotra A. Disruptions in care for Medicare beneficiaries with severe mental illness during the COVID-19 pandemic. JAMA Netw Open. 2022;5(1):e2145677. doi:10.1001/jamanetworkopen.2021.45677 35089352 PMC8800078

[13] Zhu JM, Myers R, McConnell KJ, Levander X, Lin SC. Trends in outpatient mental health services use before and during the COVID-19 pandemic. Health Aff (Millwood). 2022;41(4):573-580. doi:10.1377/hlthaff.2021.01297 35377763 PMC9056059

[14] McLaughlin CG, Wyszewianski L. Access to care: remembering old lessons. Health Serv Res. 2002;37(6):1441-1443. doi:10.1111/1475-6773.12171 12546280 PMC1464050

[15] Loera LJ, Hill LG, Evoy KE, Reveles KR. Research and scholarly methods: audit studies. J Am Coll Clin Pharm. 2023;6(5):521-527. doi:10.1002/jac5.1782

[16] Cantor J, McBain RK, Kofner A, Stein BD, Yu H. Fewer than half of US mental health treatment facilities provide services for children with autism spectrum disorder. Health Aff (Millwood). 2020;39(6):968-974. doi:10.1377/hlthaff.2019.01557 32479238 PMC7773216

[17] Tenner NL, Reddy M, Block AE. Secret shopper analysis shows getting psychiatry appointment in New *York* City is well kept secret. Community Ment Health J. 2023;59(2):290-293. doi:10.1007/s10597-022-01006-9 35840739 PMC9287131

[18] Presnall NJ, Butler GC, Grucza RA. Consumer access to buprenorphine and methadone in certified community behavioral health centers: a secret shopper study. J Subst Abuse Treat. 2022;139:108788. doi:10.1016/j.jsat.2022.108788 35534359

[19] US Census Bureau. American Community Survey. Accessed February 15, 2023. https://www.census.gov/programs-surveys/acs

[20] Beetham T, Saloner B, Wakeman SE, Gaye M, Barnett ML. Access to office-based buprenorphine treatment in areas with high rates of opioid-related mortality: an audit study. Ann Intern Med. 2019;171(1):1-9. doi:10.7326/M18-3457 31158849 PMC7164610

[21] Beetham T, Saloner B, Gaye M, Wakeman SE, Frank RG, Barnett ML. Admission practices and cost of care for opioid use disorder at residential addiction treatment programs in the US. Health Aff (Millwood). 2021;40(2):317-325. doi:10.1377/hlthaff.2020.00378 33523744 PMC8638362

[22] Bertrand M, Mullainathan S. Are Emily and Greg more employable than Lakisha and Jamal? a field experiment on labor market discrimination. Am Econ Rev. 2004;94(4):991-1013. doi:10.1257/0002828042002561

[23] 1HappyBirthday.com. Latino: Popular Baby Names. Accessed October 6, 2022. https://www.1happybirthday.com/popular_names_latino.php

[24] Fumarco L, Harrell B, Button P, Schwegman D, Dils E. Gender Identity, Race, and Ethnicity-based Discrimination in Access to Mental Health Care: Evidence from an Audit Correspondence Field Experiment. National Bureau of Economic Research. Published online December 2020. 10.1086/728931 PMC12547971 41142655

[25] Leech TGJ, Irby-Shasanmi A, Mitchell AL. “Are you accepting new patients?” a pilot field experiment on telephone-based gatekeeping and Black patients’ access to pediatric care. Health Serv Res. 2019;54(Suppl 1):234-242. doi:10.1111/1475-6773.13089 30506767 PMC6341201

[26] Cook BL, Trinh NH, Li Z, Hou SSY, Progovac AM. Trends in racial-ethnic disparities in access to mental health care, 2004-2012. Psychiatr Serv. 2017;68(1):9-16. doi:10.1176/appi.ps.201500453 27476805 PMC5895177

[27] Kirby JB, Zuvekas SH, Borsky AE, Ngo-Metzger Q. Rural residents with mental health needs have fewer care visits than urban counterparts. Health Aff (Millwood). 2019;38(12):2057-2060. doi:10.1377/hlthaff.2019.00369 31794321

[28] Alegría M, NeMoyer A, Falgàs Bagué I, Wang Y, Alvarez K. Social determinants of mental health: where we are and where we need to go. Curr Psychiatry Rep. 2018;20(11):95. doi:10.1007/s11920-018-0969-9 30221308 PMC6181118

[29] Connolly SL, Miller CJ, Gifford AL, Charness ME. Perceptions and use of telehealth among mental health, primary, and specialty care clinicians during the COVID-19 pandemic. JAMA Netw Open. 2022;5(6):e2216401. doi:10.1001/jamanetworkopen.2022.16401 35671053 PMC9175071

[30] McClellan MJ, Osbaldiston R, Wu R, . The effectiveness of telepsychology with veterans: a meta-analysis of services delivered by videoconference and phone. Psychol Serv. 2022;19(2):294-304. doi:10.1037/ser0000522 33539135

[31] Rush KL, Howlett L, Munro A, Burton L. Videoconference compared to telephone in healthcare delivery: a systematic review. Int J Med Inform. 2018;118:44-53. doi:10.1016/j.ijmedinf.2018.07.007 30153920

[32] Ebbert JO, Ramar P, Tulledge-Scheitel SM, . Patient preferences for telehealth services in a large multispecialty practice. J Telemed Telecare. 2023;29(4):298-303. doi:10.1177/1357633X20980302 33461397

[33] Chen PV, Helm A, Fletcher T, . Seeing the value of video: a qualitative study on patient preference for using video in a Veteran Affairs telemental health program evaluation. Telemed Rep. 2021;2(1):156-162. doi:10.1089/tmr.2021.0005 35720740 PMC8812285

[34] Sun CF, Correll CU, Trestman RL, . Low availability, long wait times, and high geographic disparity of psychiatric outpatient care in the US. Gen Hosp Psychiatry. 2023;84:12-17. doi:10.1016/j.genhosppsych.2023.05.012 37290263

[35] Cantor JH, McBain RK, Kofner A, Stein BD, Yu H. Availability of outpatient telemental health services in the United States at the outset of the COVID-19 pandemic. Medical Care. 2021;59(4):319-323. doi:10.1097/MLR.0000000000001512 PMC7954880 33480660

[36] Canady VA. Despite telehealth growth, disparities persist in care access. Ment Health Wkly. 2023;33(24):3-5. doi:10.1002/mhw.33681

