# Supplementary material for: Availability of Mental Telehealth Services in the US
Source: JAMA Health Forum. 2024 Feb 2;5(2):e235142. doi: 10.1001/jamahealthforum.2023.5142 (PMC10837750; doi:10.1001/jamahealthforum.2023.5142)
Supplement: Supplement 1. — eAppendix. Secret Shopper Case Profiles and Survey Questionnaires eTable 1. Comparison of Facility and County Characteristics Between Successfully Contacted and Not Contacted Facilities eTable 2. Ordinary Least Squares Regression Predicting Wait Times [file jamahealthforum-e235142-s001.pdf]

# Supplemental Online Content

Cantor J, Schuler MS, Matthews S, Kofner A, Breslau J, McBain RK. Availability of Mental Telehealth Services in the US. *JAMA Health Forum* Published online February 2, 2024. doi:10.1001/jamahealthforum.2023.5142

**eAppendix.** Secret Shopper Case Profiles and Survey Questionnaires

**eTable 1.** Comparison of Facility and County Characteristics Between Successfully Contacted and Not Contacted Facilities

**eTable 2.** Ordinary Least Squares Regression Predicting Wait Times

This supplemental material has been provided by the authors to give readers additional information about their work.

**© 2024 Cantor J et al. *JAMA Health Forum***

# eAppendix. Secret Shopper Case Profiles and Survey Questionnaires

**CASE 1: MDD + AUD**

*Background: Adult diagnosed in 2019 with major depressive disorder (MDD), who hasn't received behavioral therapy in a while, but would like to re-engage using telehealth due to feeling more depressed over the past 3 months: lack of energy, feeling hopeless, trouble sleeping, overeating, unemployed since start of COVID pandemic. They do NOT have active suicidal ideation. They are curious about receiving cognitive behavioral therapy and having their medications adjusted (they are currently taking a low dose of Prozac). They are on Medicaid but are willing to pay out of pocket if need be.*

1. For Interviewer: What is the Facility ID? ____________ (validation: always 6 numeric digits)

2. For Interviewer: Hi, my name is [NAME].
   a. Lakisha Washington (Black/Female)
   b. Jamal Jones (Black/Male)
   c. Isabella Hernandez (Hispanic/Female)
   d. Carlos García (Hispanic/Male)
   e. Emily Walsh (White/Female)
   f. Greg Baker (White/Male)

3. Are you accepting new patients right now?
   a. Yes →Great [Proceed to #4]
   b. No → I see. Do you mind if I ask a few questions about your services anyway, in case you start taking new patients in the future?
      i. No→Okay. [Hang up]
      ii. Yes→Okay. [Proceed to #4]

4. Do you take Medicaid?
   a. Yes→Okay. [Skip to #6]
   b. No→I see. [Proceed to #5]
   c. I don't know→Okay. [Skip to #6]

5. Is it only out-of-pocket payment that you accept, or do you take private insurance?
   a. Out of pocket only [Proceed to #6]
   b. Private insurance AND out of pocket [Proceed to #6]

6. Do you provide any telehealth services?
   a. Yes→I see. [Proceed to #7]
   b. No→I see. Okay, well, thank you for your time. [Hang up]
   c. I don't know→I see. Okay, well, thank you for your time. [Hang up]

7. Are telehealth services by phone or by video, or do you have both?

© 2024 Cantor J et al. *JAMA Health Forum*

a. Phone [Proceed to #8]
b. Video [Proceed to #8]
c. Both [Proceed to #8]
d. I don't know [Proceed #8]

8. Do you provide telehealth counseling / behavioral therapy for patients *with depression*?
   a. Yes → Great [Proceed to #9]
   b. Yes, but with exceptions → What exceptions? [Record response] [Proceed to #9]
   c. No → I see. What does this facility provide telehealth counseling / behavioral therapy for? [Record response] [Proceed to #9]
   d. I don't know → I see. What does this facility provide telehealth counseling / behavioral therapy for? [Record response] [Proceed to #9]

9. Do you provide medication management through telehealth for patients *with depression* (I am currently taking *Prozac*)?
   a. Yes → Great [Proceed to #10].
   b. Yes, but with exceptions → What exceptions? [Record response] [Proceed to #10]
   c. No → I see. What does this facility provide medication management for? [Record response] [Proceed to #10]
   d. I don't know → I see. What does this facility provide t medication management for? [Record response] [Proceed to #10]

10. Does this facility provide diagnostic services using telehealth? I have been previously diagnosed with *depression*, but it was several years ago.
    a. Yes → Great [Proceed to #11].
    b. Yes, but with exceptions → What exceptions? [Record response] [Proceed to #11]
    c. No → I see. What does this facility provide diagnostic services for? [Record response] [Proceed to #11]
    d. I don't know → I see. What does this facility provide diagnostic services for? [Record response] [Proceed to #11]

**Coding Note:** If response to 8, 9, and 10 are all "No", SKIP to Question 13 (because the facility doesn't offer any telehealth services for the mental health condition in question).

11. What's the soonest date that you could schedule me for a telehealth appointment?
    a. [Record response]. Okay, gotcha. [Proceed to #12]
    b. I can't give this to you right now → Okay. [Proceed to #12]
12. Are telehealth services available in languages other than English?
    a. Yes → [Record languages]. Okay. [Proceed to #13]
    b. No → Okay. [Proceed to #13]
    c. I don't know →Okay. [Proceed to #13]
13. Also, do you provide telehealth counseling / behavioral therapy for people who want to work on their *drinking*?
    a. Yes→Great [Proceed to #14]
    b. No→I see. [Proceed to #14]

© 2024 Cantor J et al. *JAMA Health Forum*

    c. I don't know→I see. [Proceed to #14]

14. Does this facility provide medication treatment for *alcohol problems* through telehealth?
    a. Yes→Okay. [Proceed to #15]
    b. No→ Okay. [Proceed to #15]
    c. I don't know→ Okay. [Proceed to #15]
15. Does this facility provide diagnostic services for *alcohol problems* through telehealth? I'm not sure if I really have a problem with *alcohol* or if I'm just *depressed*.
    a. Yes→Okay. [Proceed to #16]
    b. No→ Okay. [Proceed to #16]
    c. I don't know→ Okay. [Proceed to #16]
16. Would the same treatment provider be able to work with me on both my *depression and alcohol*, or would I have to see different providers?
    a. Concurrent treatment with one provider →I see. [Proceed to #17]
    b. Two separate providers →I see. [Proceed to #17]
    c. Sometimes the same, sometimes different → I see. [Proceed to #17]
    d. I don't know→Okay. [Proceed to #17]
17. One last question: do you offer telehealth services to patients residing in other states?
    a. Yes → Okay. Before I move forward with this, I want to take some time to think about your answers, but I really appreciate your time! [Hang up]
    b. No → Okay. Before I move forward with this, I want to take some time to think about your answers, but I really appreciate your time! [Hang up]
    c. I don't know → Okay. Before I move forward with this, I want to take some time to think about your answers, but I really appreciate your time! [Hang up]
    d. Which state are you in? [Provide a response that is a neighboring state]. Before I move forward with this, I want to take some time to think about your answers, but I really appreciate your time! [Hang up]

18. If you have any qualitative notes you want to document from your conversation, please list here.

[SUBMIT]

© 2024 Cantor J et al. *JAMA Health Forum*

## CASE 2: GAD + AUD, LOOKING TO RE-ENGAGE IN CBT/ADT

*Background: Adult diagnosed in 2019 with generalized anxiety disorder (GAD), who hasn't received behavioral therapy in a while, but would like to re-engage using telehealth due to feeling more anxious over the past 3 months: unexplained panic attacks that are accompanied by physical distress—including restlessness, feeling on edge, difficulty concentrating, muscle tension, trouble falling asleep, teeth clenching. They have been feeling low energy/down, but they attribute it to the impairment from their anxiety. They are curious about receiving cognitive behavioral therapy and having their medications adjusted (they are currently taking a low dose of Lexapro). They are on Medicaid but are willing to pay out of pocket if need be.*

1. For Interviewer: What is the Facility ID? ____________ (validation: always 6 numeric digits)

2. For Interviewer: Hi, my name is [NAME].
    a. Lakisha Washington (Black/Female)
    b. Jamal Jones (Black/Male)
    c. Isabella Hernandez (Hispanic/Female)
    d. Carlos García (Hispanic/Male)
    e. Emily Walsh (White/Female)
    f. Greg Baker (White/Male)

3. Are you accepting new patients right now?
    a. Yes →Great [Proceed to #4]
    b. No → I see. Do you mind if I ask a few questions about your services anyway, in case you start taking new patients in the future?
        i. No→Okay. [Hang up]
        ii. Yes→Okay. [Proceed to #4]

4. Do you take Medicaid?
    a. Yes→Okay. [Skip to #6]
    b. No→I see. [Proceed to #5]
    c. I don't know→Okay. [Skip to #6]

5. Is it only out-of-pocket payment that you accept, or do you take private insurance?
    a. Out of pocket only [Proceed to #6]
    b. Private insurance AND out of pocket [Proceed to #6]

6. Do you provide any telehealth services?
    a. Yes→I see. [Proceed to #7]
    b. No→I see. Okay, well, thank you for your time. [Hang up]
    c. I don't know→I see. Okay, well, thank you for your time. [Hang up]

7. Are telehealth services by phone or by video, or do you have both?
    a. Phone [Proceed to #8]
    b. Video [Proceed to #8]

© 2024 Cantor J et al. *JAMA Health Forum*

    c. Both [Proceed to #8]
    d. I don't know [Proceed #8]

8. Do you provide telehealth counseling / behavioral therapy for patients *with anxiety*?
    a. Yes → Great [Proceed to #9]
    b. Yes, but with exceptions → What exceptions? [Record response] [Proceed to #9]
    c. No → I see. What does this facility provide telehealth counseling / behavioral therapy for? [Record response] [Proceed to #9]
    d. I don't know → I see. What does this facility provide telehealth counseling / behavioral therapy for? [Record response] [Proceed to #9]

9. Do you provide medication management through telehealth for patients *with anxiety* (I am currently taking *Lexapro*)?
    a. Yes → Great [Proceed to #10].
    b. Yes, but with exceptions → What exceptions? [Record response] [Proceed to #10]
    c. No → I see. What does this facility provide medication management for? [Record response] [Proceed to #10]
    d. I don't know → I see. What does this facility provide t medication management for? [Record response] [Proceed to #10]

10. Does this facility provide diagnostic services using telehealth? I have been previously diagnosed with *generalized anxiety disorder*, but it was several years ago.
    a. Yes → Great [Proceed to #11].
    b. Yes, but with exceptions → What exceptions? [Record response] [Proceed to #11]
    c. No → I see. What does this facility provide diagnostic services for? [Record response] [Proceed to #11]
    d. I don't know → I see. What does this facility provide diagnostic services for? [Record response] [Proceed to #11]

**Coding Note:** If response to 8, 9, and 10 are all "No", the caller should thank the provider for their time and then SKIP to Question 13 (because the facility doesn't offer any telehealth services for the mental health condition in question).

11. What's the soonest date that you could schedule me for a telehealth appointment?
    a. [Record response]. Okay, gotcha. [Proceed to #12]
    b. I can't give this to you right now → Okay. [Proceed to #12]
12. Are telehealth services available in languages other than English?
    a. Yes → [Record languages]. Okay. [Proceed to #13]
    b. No → Okay. [Proceed to #13]
    c. I don't know →Okay. [Proceed to #13]
13. Also, do you provide telehealth counseling / behavioral therapy for people who want to work on their *drinking*?
    d. Yes→Great [Proceed to #14]
    e. No→I see. [Proceed to #14]
    f. I don't know→I see. [Proceed to #14]

© 2024 Cantor J et al. *JAMA Health Forum*

14. Does this facility provide medication treatment for *alcohol problems* through telehealth?
    a. Yes→Okay. [Proceed to #15]
    b. No→ Okay. [Proceed to #15]
    c. I don't know→ Okay. [Proceed to #15]
15. Does this facility provide diagnostic services for *alcohol problems* through telehealth? I'm not sure if I really have a problem with *alcohol* or if I'm just *anxious*.
    a. Yes→Okay. [Proceed to #16]
    b. No→ Okay. [Proceed to #16]
    c. I don't know→ Okay. [Proceed to #16]
16. Would the same treatment provider be able to work with me on both my *anxiety and alcohol use*, or would I have to see different providers?
    a. Concurrent treatment with one provider →I see. [Proceed to #17]
    b. Two separate providers →I see. [Proceed to #17]
    c. Sometimes the same, sometimes different → I see. [Proceed to #17]
    d. I don't know→Okay. [Proceed to #17]
17. One last question: do you offer telehealth services to patients residing in other states?
    e. Yes → Okay. Before I move forward with this, I want to take some time to think about your answers, but I really appreciate your time! [Hang up]
    f. No → Okay. Before I move forward with this, I want to take some time to think about your answers, but I really appreciate your time! [Hang up]
    g. I don't know → Okay. Before I move forward with this, I want to take some time to think about your answers, but I really appreciate your time! [Hang up]
    h. Which state are you in? [Provide a response that is a neighboring state]. Before I move forward with this, I want to take some time to think about your answers, but I really appreciate your time! [Hang up]

18. If you have any qualitative notes you want to document from your conversation, please list here.

[SUBMIT]

**CASE 3: SZ + AUD, LOOKING TO RE-ENGAGE IN CBT/ADT**

*Background: Adult diagnosed in 2019 with schizophrenia (SZ), who hasn't received counseling/behavioral therapy in a while, but would like to re-engage using telehealth. Their symptom profile includes: paranoia (particularly that friends/family members are 'out to get' them), trouble keeping their thoughts straight, difficulties with self-care, social isolation. When they aren't on their meds (which they currently are), they have occasionally heard voices that other people haven't. They are curious about receiving cognitive behavioral therapy and having their medications adjusted (they are currently taking Risperdal at 4 mg/day). They are on Medicaid but are willing to pay out of pocket if need be.*

1. For Interviewer: What is the Facility ID? ____________ (validation: always 6 numeric digits)

2. For Interviewer: Hi, my name is [NAME].
   a. Lakisha Washington (Black/Female)
   b. Jamal Jones (Black/Male)
   c. Isabella Hernandez (Hispanic/Female)
   d. Carlos García (Hispanic/Male)
   e. Emily Walsh (White/Female)
   f. Greg Baker (White/Male)

3. Are you accepting new patients right now?
   a. Yes →Great [Proceed to #4]
   b. No → I see. Do you mind if I ask a few questions about your services anyway, in case you start taking new patients in the future?
      i. No→Okay. [Hang up]
      ii. Yes→Okay. [Proceed to #4]

4. Do you take Medicaid?
   a. Yes→Okay. [Skip to #6]
   b. No→I see. [Proceed to #5]
   c. I don't know→Okay. [Skip to #6]

5. Is it only out-of-pocket payment that you accept, or do you take private insurance?
   a. Out of pocket only [Proceed to #6]
   b. Private insurance AND out of pocket [Proceed to #6]

6. Do you provide any telehealth services?
   a. Yes→I see. [Proceed to #7]
   b. No→I see. Okay, well, thank you for your time. [Hang up]
   c. I don't know→I see. Okay, well, thank you for your time. [Hang up]

7. Are telehealth services by phone or by video, or do you have both?
   a. Phone [Proceed to #8]
   b. Video [Proceed to #8]
   c. Both [Proceed to #8]
   d. I don't know [Proceed #8]

© 2024 Cantor J et al. *JAMA Health Forum*

8. Do you provide telehealth counseling / behavioral therapy for patients *schizophrenia*?
    a. Yes → Great [Proceed to #9]
    b. Yes, but with exceptions → What exceptions? [Record response] [Proceed to #9]
    c. No → I see. What does this facility provide telehealth counseling / behavioral therapy for? [Record response] [Proceed to #9]
    d. I don't know → I see. What does this facility provide telehealth counseling / behavioral therapy for? [Record response] [Proceed to #9]

9. Do you provide medication management through telehealth for patients *with schizophrenia* (I am currently taking *Risperidone*)?
    a. Yes → Great [Proceed to #10].
    b. Yes, but with exceptions → What exceptions? [Record response] [Proceed to #10]
    c. No → I see. What does this facility provide medication management for? [Record response] [Proceed to #10]
    d. I don't know → I see. What does this facility provide t medication management for? [Record response] [Proceed to #10]

10. Does this facility provide diagnostic services using telehealth? I have been previously diagnosed with *schizophrenia*, but it was several years ago.
    a. Yes → Great [Proceed to #11].
    b. Yes, but with exceptions → What exceptions? [Record response] [Proceed to #11]
    c. No → I see. What does this facility provide diagnostic services for? [Record response] [Proceed to #11]
    d. I don't know → I see. What does this facility provide diagnostic services for? [Record response] [Proceed to #11]

**Coding Note:** If response to 8, 9, and 10 are all "No", the caller should thank the provider for their time and then SKIP to Question 13 (because the facility doesn't offer any telehealth services for the mental health condition in question).

11. What's the soonest date that you could schedule me for a telehealth appointment?
    a. [Record response]. Okay, gotcha. [Proceed to #12]
    b. I can't give this to you right now → Okay. [Proceed to #12]
12. Are telehealth services available in languages other than English?
    a. Yes → [Record languages]. Okay. [Proceed to #13]
    b. No → Okay. [Proceed to #13]
    c. I don't know →Okay. [Proceed to #13]
13. Also, do you provide telehealth counseling / behavioral therapy for people who want to work on their *drinking*?
    g. Yes→Great [Proceed to #14]
    h. No→I see. [Proceed to #14]
    i. I don't know→I see. [Proceed to #14]
14. Does this facility provide medication treatment for *alcohol problems* through telehealth?
    a. Yes→Okay. [Proceed to #15]

© 2024 Cantor J et al. *JAMA Health Forum*

  b. No→ Okay. [Proceed to #15]
  c. I don't know→ Okay. [Proceed to #15]
15. Does this facility provide diagnostic services for *alcohol problems* through telehealth? I'm not sure if I really have a problem with *alcohol* or if it's just my schizophrenia.
  a. Yes→Okay. [Proceed to #16]
  b. No→ Okay. [Proceed to #16]
  c. I don't know→ Okay. [Proceed to #16]
16. Would the same treatment provider be able to work with me on both my *schizophrenia and alcohol use*, or would I have to see different providers?
  a. Concurrent treatment with one provider →I see. [Proceed to #17]
  b. Two separate providers →I see. [Proceed to #17]
  c. Sometimes the same, sometimes different → I see. [Proceed to #17]
  d. I don't know→Okay. [Proceed to #17]
17. One last question: do you offer telehealth services to patients residing in other states?
  i. Yes → Okay. Before I move forward with this, I want to take some time to think about your answers, but I really appreciate your time! [Hang up]
  j. No → Okay. Before I move forward with this, I want to take some time to think about your answers, but I really appreciate your time! [Hang up]
  k. I don't know → Okay. Before I move forward with this, I want to take some time to think about your answers, but I really appreciate your time! [Hang up]
  l. Which state are you in? [Provide a response that is a neighboring state]. Before I move forward with this, I want to take some time to think about your answers, but I really appreciate your time! [Hang up]

18. If you have any qualitative notes you want to document from your conversation, please list here.

[SUBMIT]

© 2024 Cantor J et al. *JAMA Health Forum*

**eTable 1:** Comparison of Facility and County Characteristics Between Successfully Contacted and Not Contacted Facilities (n=7,089)

| Characteristic of Interest | Facilities Successfully Contacted (n=1,404) | | Facilities Not Contacted (n=5,685) | |
|---|---|---|---|---|
| | n | % | n | % |
| **Facility Characteristics** | | | | |
| *Services offered* | | | | |
| Outpatient services only | 1,277 | 91.0% | 5,066 | 89.1% |
| *Ownership* | | | | |
| Government | 198 | 14.1% | 698 | 12.3% |
| Private, Por-Profit | 319 | 22.7% | 1,372 | 24.1% |
| Private, Not-for-Profit | 887 | 63.2% | 3,615 | 63.6% |
| Community Mental Health Center | 376 | 26.8% | 1,365 | 24.0% |
| Accepts Medicaid | 1,290 | 91.9% | 5,175 | 91.0% |
| Accepts Private Insurance | 1,236 | 88.0% | 4,847 | 85.3% |
| **County Characteristics** | | | | |
| *Rurality* | | | | |
| Not Metropolitan | 385 | 27.4% | 1,413 | 24.9% |
| Metropolitan | 1,019 | 72.6% | 4,272 | 75.2% |
| *Share of Hispanic Residents* | | | | |
| <5% | 473 | 33.7% | 1,719 | 30.2% |
| 5-10% | 336 | 23.9% | 1,340 | 23.6% |
| 10-20% | 294 | 20.9% | 1,240 | 21.8% |
| >20% | 301 | 21.4% | 1,386 | 24.4% |
| *Share of non-Hispanic Black/African American Residents* | | | | |
| <5% | 626 | 44.6% | 2,613 | 46.0% |
| 5-10% | 274 | 19.5% | 1,082 | 19.0% |
| 10-20% | 230 | 16.4% | 979 | 17.2% |
| >20% | 274 | 19.5% | 1,011 | 17.8% |

© **2024 Cantor J et al. *JAMA Health Forum***

| | | | | |
|---|---|---|---|---|
| *Median Household Income* | | | | |
| Below Median ($58,235) | 439 | 31.3% | 1,486 | 26.1% |
| Above Median | 965 | 68.7% | 4,199 | 73.9% |
| *Share of Residents 25 years or older with High School Degree* | | | | |
| Below Median (89.2%) | 624 | 44.4% | 2,296 | 40.4% |
| Above Median | 780 | 55.6% | 3,389 | 59.6% |
| **Caller Characteristics** | | | | |
| *Presenting Condition* | | | | |
| General Anxiety Disorder | 477 | 34.0% | N/A | N/A |
| Major Depressive Disorder | 450 | 32.1% | N/A | N/A |
| Schizophrenia | 477 | 34.0% | N/A | N/A |
| *Inferred Race and Ethnicity* | | | N/A | N/A |
| Hispanic | 459 | 32.7% | N/A | N/A |
| Non-Hispanic White | 457 | 32.6% | N/A | N/A |
| Non-Hispanic Black | 488 | 34.8% | N/A | N/A |

**Notes:** Facility characteristics come from a data download of the MATTR dataset on August 22, 2022. County-level characteristics come from the 2020 American Community Survey. Facilities that were outpatient only, operated by the U.S. Department of Veteran Affairs, and facilities with multiple co-located treatment programs were all excluded.

© 2024 Cantor J et al. *JAMA Health Forum*

**eTable 2: Ordinary Least Squares Regression Predicting Wait Times**

| | |
|---|---|
| **Caller Characteristics** | |
| *Presenting Condition (Ref = Generalized Anxiety Disorder)* | |
| Major Depressive Disorder | -2.795 |
| | (4.416) |
| Schizophrenia | 4.160 |
| | (3.827) |
| *Inferred Race and Ethnicity (Ref = Non-Hispanic White)* | |
| Hispanic | 6.266 |
| | (5.371) |
| Black | 4.216 |
| | (3.694) |
| **Facility Characteristics** | |
| Accepts Medicaid as a form of payment | 13.01 |
| | (7.518) |
| Accepts private insurance as a form of payment | -4.723 |
| | (4.044) |
| Community Mental Health Center | -7.528 |
| | (4.037) |
| *Ownership (Ref = Government)* | |
| Private, for-profit | -3.532 |
| | (8.695) |
| Private, not-for-profit | 7.331 |
| | (7.614) |
| Outpatient only facility (*Ref* = not outpatient only facility) | -6.785 |
| | (7.496) |
| **County Characteristics** | |
| Metropolitan (*Ref* = not Metropolitan) | 3.997 |

© **2024 Cantor J et al. *JAMA Health Forum***

| | |
|---|---|
| | (5.954) |
| *Share of Hispanic Residents (Ref = <5%)* | |
| 5-10% | -0.359 |
| | (6.244) |
| 10-20% | 7.509 |
| | (9.363) |
| >20% | 2.354 |
| | (12.47) |
| *Share of non-Hispanic Black/African American Residents (Ref = <5%)* | |
| 5-10% | 4.844 |
| | (5.954) |
| 10-20% | 8.490 |
| | (5.992) |
| >20% | -3.046 |
| | (6.774) |
| Above median household income (*Ref* = below median) | 9.017 |
| | (4.650) |
| Above median % of residents with a high school degree (*Ref* = below median) | 0.811 |
| | (4.495) |

**Note:** Ordinary least squares regression was estimated that also included state fixed effects. Standard errors are in parentheses. Standard errors were clustered at the state-level. *** p<0.001, ** p<0.01, * p<0.05

© 2024 Cantor J et al. *JAMA Health Forum*
