# Supplementary material for: Availability of Mental Telehealth Services in the US
Source: JAMA Health Forum. 2024 Feb 2;5(2):e235142. doi: 10.1001/jamahealthforum.2023.5142 (PMC10837750; doi:10.1001/jamahealthforum.2023.5142)
Supplement: Supplement 2. — Data Sharing Statement [file jamahealthforum-e235142-s002.pdf]

Data Sharing Statement

Cantor. Availability of Mental Telehealth Services in the US. *JAMA Health Forum*. Published February 02, 2024. doi:10.1001/jamahealthforum.2023.5142

Data
Data available: No

Additional Information
Explanation for why data not available: We will provide aggregated data but at risk of disclosure not individual provider data.
